# Environmental Tobacco Smoke Exposure Estimated Using the SHSES Scale, and Feature Tracking Computed Tomography-Derived Left Ventricular Global Longitudinal Strain in Hypertensive Patients

**DOI:** 10.1007/s12012-022-09770-6

**Published:** 2022-10-31

**Authors:** Paweł Gać, Adrian Martuszewski, Patrycja Paluszkiewicz, Małgorzata Poręba, Grzegorz Mazur, Rafał Poręba

**Affiliations:** 1 Division of Environmental Health and Occupational Medicine, Department of Population Health, Wroclaw Medical University, Mikulicza-Radeckiego 7, 50-368 Wrocław, Poland; 2 Centre of Diagnostic Imaging, 4th Military Hospital, Weigla 5, 50-981 Wrocław, Poland; 3 Department of Emergency Medical Service, Wroclaw Medical University, 50-367 Wrocław, Poland; 4 Department of Paralympic Sports, Wroclaw University of Health and Sport Sciences, Witelona 25a, 51-617 Wrocław, Poland; 5 Department of Internal Medicine, Occupational Diseases and Hypertension, Wroclaw Medical University, Borowska 213, 50-556 Wrocław, Poland

**Keywords:** Computed tomography, Environmental tobacco smoke, Global longitudinal strain, Left ventricular, SHSES scale

## Abstract

Aim of the study was to assess the relationship between environmental tobacco smoke (ETS) and computed tomography-derived left ventricular global longitudinal strain (LV GLS) in patients with arterial hypertension. 103 non-smokers with AH were included in the study (age 67.73 ± 8.84 years). ETS exposure was assessed with the Second-Hand Smoke Exposure Scale (SHSES). LV GLS was measured on computed tomography using feature tracking technology. In accordance with SHSES scale patients were divided into subgroups: subgroup A—no ETS exposure, subgroup B—low ETS exposure, subgroup C—medium ETS exposure, and subgroup D—high ETS exposure. Peak of LV GLS was statistically significantly lower in subgroup D than in subgroup A. There was a negative correlation between the exposure to ETS expressed by the SHSES scale and peak of LV GLS (*r* = − 0.35, *p* < 0.05). Regression analysis showed that higher SHSES score, higher age, left ventricular hypertrophy, left ventricular diastolic dysfunction, and higher CAD-RADS are independent risk factors for lower peak of LV GLS values. On the contrary, the effective blood pressure control appeared to be independent protecting factor against lower peak of LV GLS values. In summary, there is an unfavorable weak relationship between ETS exposure estimated using the SHSES scale and LV GLS in hypertensive patients.

## Introduction

Since 1990, there is a twofold increase in the number of cases of arterial hypertension (AH). AH is responsible for over 8 million deaths from stroke, ischemic heart disease, and others [1]. Tobacco smokers have a greater risk of AH development. It is due to arterial stiffness and activation of inflammatory processes, and increased production of reactive oxygen species (ROS) resulting in destructive impact on vascular wall and dysfunction of endothelium. Smoking and AH have a synergistic effect on the risk of stroke [2]. Tobacco smoking has an impact on several human organs, especially on cardiovascular system. It can cause cardiac remodeling, especially resulting in left ventricular (LV) dysfunction and its hypertrophy [3].

Environmental tobacco smoke (ETS) is a health hazard which includes second-hand smoking (SHS) and passive smoking. The degree of ETS exposure can be expressed using the Second-Hand Smoke Exposure Scale (SHSES) validated by Vardavas et al. [4]. The scale is a questionnaire which consists of 11 points related to ETS exposure in work, home, car, and public places. Its interpretation and scoring are presented in Table 1. There is also a relationship between ETS and AH. Similarly, as mentioned above active smokers as well as passive smokers have dysfunction of endothelium, greater stiffness of arteries and have increased risk of heart diseases [5]. Study conducted on rabbits exposed to tobacco smoking showed that ETS in these animals can cause LV hypertrophy [6].

**Table 1:** The Second-Hand Smoke Exposure Scale and its interpretation

Type of exposure	Conditions (scoring)	SHSES
Exposure at home (per day)	> 20 cigarettes (5)	0—no ETS exposure 1–3—low ETS exposure 4–7—medium ETS exposure 8–11—high ETS exposure
	10–20 cigarettes (4)	
	6–9 cigarettes (3)	
	1–5 cigarettes (2)	
	None (0)	
Exposure in car (per day)	30 min or more (3)	
	Less than 30 min (2)	
	Never (0)	
Exposure in public places (past week)	Once or more (2)	
	Never (0)	
Exposure at work (past week)	Once or more (1)	
	Never (0)	

*ETS* environmental tobacco smoke, *SHSES* Second-Hand Smoke Exposure Scale

A measure of tissue deformation is strain, which is defined as the change in length normalized to the original length. Strain rate describes the rate at which this change occurs. The strain is accurate in defining regional diastolic and systolic function in radial, circumferential, longitudinal, or torsional directions [7]. Strain can be assessed by echocardiography (ECHO) and have a utility in prognosing patients with coronary artery disease (CAD), mitral stenosis, and other cardiac diseases [8]. Next to ECHO, it is possible to measure strain using magnetic resonance imaging (MRI) [9] and computed tomography (CT) [10, 11]. Global longitudinal strain (GLS) has better prognostic value in predicting adverse cardiac events than left ventricular ejection fraction (LVEF) measurements. Obtaining strain measurements using cardiac CT have high repeatability and does not depend on external factors [11, 12]. Left ventricular global longitudinal strain (LV GLS) measurements are independent predictor for reverse remodeling of LV and for patients with sinus rhythm the optimal cut off value is − 10% [13]. There is also possibility of assessing maximum principal strain (MP strain, which measures three-dimensional maximum extension size) but it does not correlate with global radial strain (GRS) and global circumferential strain (GCS) assessed by speckle tracking echocardiography (STE) [12].

The aim of the study was to assess the relationship between ETS and feature tracking computed tomography-derived LV GLS in patients with AH.

## Methods

For this cross-sectional study, 103 non-smokers with AH were included. The group consisted of 49 females and 54 males with the mean age of 67.73 ± 8.84 years. Inclusion criteria were age ≥ 18, arterial hypertension pharmacologically treated ≥ 5 years, no history of smoking cigarettes , and indication to coronary CT angiography. The exclusion criteria were insufficient quality of the coronary CT angiography, secondary hypertension, previously diagnosed ischemic heart disease, previous stroke, type 2 diabetes, chronic kidney disease, and hyperthyroidism or hypothyroidism. Group characteristics are presented in Table 2.

**Table 2:** Clinical characteristics of the study group (*n* = 103)

Age (years)^b^	67.73 ± 8.84
Gender	
Men^a^	48.5
Women^a^	51.5
Height (cm)^b^	167.61 ± 8.08
Body mass (kg)^b^	72.96 ± 11.75
BMI (kg/m^2^)^b^	25.97 ± 3.32
Essential hypertension^a^	100.0
Systolic blood pressure (mmHg)^b^	138.74 ± 15.27
Diastolic blood pressure (mmHg)^b^	85.58 ± 10.01
Effective blood pressure control^a^	51.4
Hypotensive drugs	
ACE inhibitors^a^	59.2
β-blockers^a^	42.7
Diuretics^a^	28.1
Calcium channel blockers^a^	30.1
Angiotensin receptor blockers^a^	9.7
Lipid profile	
Total cholesterol (mg/dl)^b^	224.01 ± 49.03
LDL cholesterol (mg/dl)^b^	116.96 ± 29.34
HDL cholesterol (mg/dl)^b^	55.03 ± 18.94
Triglycerides (mg/dl)^b^	188.65 ± 169.241
Fasting glucose (mg/dl)^b^	110.06 ± 41.29
Indication to CCTA^a^	
CAD suspicion	70.9
Chest pain	29.1
Low intermediate CAD risk	29.1
Numerous CAD risk factors	19.4
Inconclusive or non-diagnostic exercise test	16.5
Regional wall motion abnormalities of left ventricular	14.6
Sudden cardiac death in the family history	1.9

*ACE* angiotensin-converting enzyme, *CAD* coronary artery diseases, *CCTA* coronary computed tomography angiography, *BMI* body mass index, *HDL* high density lipoprotein, *LDL* low density lipoprotein

^a^Percentages

^b^Arithmetic mean ± standard deviation

The International Review Board (IRB) approval for this study was obtained. Study was performed in accordance with Good Clinical Practise (GCP) principles. Written informed consent was also obtained from all participants.

The study protocol assumed: taking medical history, obtaining anthropometric features, collecting of blood samples to perform some laboratory blood tests, assessment in accordance with SHSES scale, transthoracic echocardiography, and coronary CT angiography.

Study participants had several blood parameters assessed: total cholesterol concentration, LDL cholesterol, HDL cholesterol, triglycerides, and glucose. Lipid profile and fasting glucose concentration were measured using standard methods described in the instructions provided with the ordered reagent kits. The blood pressure was measured in accordance with clinical guidelines by Korotkov method. The effectiveness of blood pressure control was assessed based on the 24-h ambulatory blood pressure monitoring (ABPM) performed in the last 6 months.

ETS exposure was assessed with SHSES. In accordance with SHSES scale, patients were divided into subgroups: subgroup A—no ETS exposure (SHSES = 0 points, *n* = 48), subgroup B—low ETS exposure (SHSES = 1–3 points, *n* = 16), subgroup C—medium ETS exposure (SHSES = 4–7 points, *n* = 21), and subgroup D—high ETS exposure (SHSES = 8–11 points, *n* = 18).

Transthoracic echocardiography was performed using the standard method. Using M-mode, left ventricular end-diastolic diameter (LVEDD), left ventricular end-systolic diameter (LVESD), interventricular septum end-diastolic diameter (IVSEDD), and posterior wall end-diastolic diameter (PWEDD) were measured. Left ventricular ejection fraction (LVEF) was determined from the apical 4-chamber and 2-chamber view, with the biplane Simpson’s method. Left ventricular mass (LVM), left ventricular mass index (LVMI), and relative wall thickness (RWT) were calculated. Left ventricular hypertrophy (LVH) was diagnosed when RWT > 0.45 or LVMI > 125 g/m^2^ in men and 110 g/m^2^ in women. The left ventricular diastolic function parameters were determined by pulsed Doppler and tissue Doppler techniques. Using pulsed Doppler maximum early diastolic mitral flow velocity (*E*), maximum late diastolic mitral flow velocity (*A*), deceleration time of *E* velocity (DT), and isovolumetric relaxation time (IVRT) were evaluated. Using tissue Doppler, early diastolic mitral annular velocity (*E*′) was evaluated. Left ventricular diastolic dysfunction (LVDD) was diagnosed when *E*/*E*′ ratio ≥ 15. When the value of *E*/*E*′ ratio was within the range of 8 to 15 accessory criteria were employed, based on evaluation of mitral inflow profile, left atrium volume index (LAVI), and left ventricular mass index (LVMI). LVDD was diagnosed when *E*/*A* ratio < 0.5 and DT > 280 ms or LAVI > 40 ml/m^2^ or LVMI > 122 g/m^2^ for females or LVMI > 149 g/m^2^ for men. Echocardiography was performed in all patients by a cardiologist with 20 years of experience.

The cardiac computed tomography (CCT) was performed using the standard coronary CT angiography (CCTA) protocol with dual-source 128-slice CT scanner SOMATOM Definition Dual-Source CT (Siemens Healthcare, Erlangen, Germany). The coronary artery disease severity was determined based on the Coronary Artery Disease–Reporting and Data System (CAD-RADS), where 0—documented absence of coronary artery disease (CAD), 1—minimal non-obstructive CAD (maximal stenosis: 1–24%), 2—mild non-obstructive CAD (maximal stenosis: 25–49%), 3—moderate CAD (maximal stenosis: 50–69%), 4—severe CAD (maximal stenosis: 70–99%), and 5—total coronary artery occlusion.

The original CT images, obtained by means of retrospective ECG gating for the entire heart cycle, were processed using Medis Suite 3.2 software (Medis, Leiden, Netherlands) into 2D cinematographic loops of 3 LAX cardiological projections, i.e., 2-chamber, 3-chamber, and 4-chamber. The following reconstruction parameters were used: slice thickness of 0.6 mm, a reconstruction increment of 0.4 mm, and temporal resolution of 10 phases per cardiac cycle in 10% increments from early systole (0% cardiac cycle) to end-diastole (90% cardiac cycle). The obtained secondary CINE reconstructions were used for the planimetric assessment of the left ventricular volume. The following volume parameters were calculated: left ventricular end-diastolic volume (LVEDV), left ventricular end-systolic volume (LVESV), and left ventricular stroke volume (LVSV). Based on the “LVSV/LVEDV*100%” formula, the left ventricular ejection fraction (LVEF) was calculated.

The obtained secondary cinematographic reconstructions were also used for the assessment of LV GLS. Using the Medis Suite LV Strain application (Medis, Leiden, Netherlands), the feature tracking method, the parameters of the left ventricular myocardial longitudinal strain (LS) were calculated. Strain analysis was performed semi-automatically with manual correction. In the images along the short axis, points corresponding to the endocardium and epicardium were marked in each of the three locations in the basal-ventricular, mid-ventricular, and apex-ventricular layers. Based on the indicated points, the application drew outlines of the endocardium and epicardium, which were then analyzed and accepted or manually corrected by the researcher. The papillary muscle was considered part of the left ventricular lumen.

Peak LS values were assessed for each of 16 segments of the left ventricle: 6 basal segments, 6 mid-chamber segments, and 4 apical segments. In the basal and mid-chamber layer, the anterior, antero-septal, infero-septal, inferior, infero-lateral, and antero-lateral segments were analyzed. By contrast, in the apical layer, the anterior, septal, inferior, and lateral segments were analyzed. Then, peak LS was assessed for the whole basal, mid-chamber, and apical layer. Global longitudinal strain (GLS) was assessed by averaging the peak strain values of 16 segments extracted from three LAX projections.

Strain analysis was performed by one cardiovascular radiologist with 10 years of experience in cardiac CT evaluation, passed European Association of Cardiovascular Imaging cardiac computed tomography exam and national certification in the field of cardiovascular radiology research. For the reproducibility of the GLS assessment, a 10% group of 11 CCT studies was randomized in which the researcher re-analyzed the strain more than 6 weeks after the previous assessment. In this group of CCT studies, the strain was also assessed by a second researcher—a cardiologist with 20 years of echocardiographic experience and 5 years of experience in CCT evaluation. Strain analysis results were highly reproducible. The intra-observer reproducibility parameters were coefficient of variance 4.1%, intra-class correlation coefficient 0.98. On the other hand, the inter-observer reproducibility parameters amounted to coefficient of variance 7.3%, intra-class correlation coefficient 0.92.

Statistical analysis was performed with the application “Dell Statistica” (Dell Inc., USA). Quantitative variables were presented as arithmetic means ± standard deviations. The distribution of variables was determined by the Shapiro–Wilk test. For normally distributed quantitative variables, the t-test or ANOVA was used to test the hypotheses. For quantitative variables with no normal distribution, the Mann–Whitney *U* test or the Kruskal–Wallis ANOVA test were used to test the hypotheses. Qualitative variables were presented as percentages. The *χ*^2^ test was used in the comparative analysis of qualitative variables. To establish the relationship between the studied variables, a correlation analysis and regression analysis were performed. The results at the level of *p* < 0.05 were considered statistically significant.

## Results

In the study group, the mean SHSES value was 33.37 ± 3.96. The minimum SHSES value of 0 (meaning no exposure to ETS) was found in 48 patients, while the maximum SHSES value of 11 was observed in 4 patients. 55 patients (53.4%) were exposed to ETS, of which 16 patients (15.5%) had low exposure, 21 patients (20.4%) had medium exposure, and 18 patients (17.5%) had high exposure.

In the study group, effective blood pressure control assessed based on the ABPM performed in the last 6 months was observed in 51.4% of patients, Table 2. In echocardiography, LVEF was 62.06 ± 7.43%, LVH was diagnosed in 46.6% of patients, and LVDD in 38.8% of patients, Table 3.

**Table 3:** Echocardiographic parameters in the group (*n* = 103)

LVEDD (mm)^b^	53.56 ± 5.48
LVESD (mm)^b^	40.16 ± 4.18
IVSEDD (mm)^b^	12.94 ± 1.42
PWEDD (mm)^b^	11.95 ± 1.03
LVMI (g/m^2^)^b^	135.14 ± 26.02
LVH^a^	46.6
LVEF (%)^b^	62.06 ± 7.43
*E*/*A*^b^	1.14 ± 0.22
DT (ms)^b^	174.27 ± 32.14
IVRT (ms)^b^	111.29 ± 21.84
*E*′ (cm/s)^b^	10.63 ± 1.20
*E*/*E*′^b^	9.74 ± 1.24
LVDD^a^	38.8

*DT* deceleration time of E velocity, *E/A* maximal early diastolic mitral flow velocity/maximal late diastolic mitral flow velocity, *E*′ early diastolic mitral annular velocity, *E/E*′ maximal early diastolic mitral flow velocity/early diastolic mitral annular velocity, *IVRT* isovolumetric relaxation time, *IVSEDD* interventricular septum end-diastolic diameter, *LVDD* left ventricular diastolic dysfunction, *LVEDD* left ventricular end-diastolic diameter, *LVEF* left ventricular ejection fraction, *LVESD* left ventricular end-systolic diameter, *LVH *left ventricular hypertrophy, *LVMI* left ventricular mass index, *PWEDD* posterior wall end-diastolic diameter

^a^Percentages

^b^Arithmetic mean ± standard deviation

Based on the obtained CCTA images, coronary artery disease was excluded in 16.5% of patients. 59.2% of patients had non-obstructive CAD (18.4% CAD-RADS 1 and 40.8% CAD-RADS 2). The coronary stenosis requiring further diagnosis was visualized in 24.2% of patients (16.5% CAD-RADS 3, 6.8% CAD-RADS 4, and 0.9% CAD-RADS 5). The assessed parameters of the CCTA test in the studied group of patients are summarized in Table 4.

**Table 4:** CCTA parameters in the study group (*n* = 103)

Heart rate during CCTA acquisition (bpm)^b^	74.16 ± 10.53
CAD-RADS^a^	
0: documented absence of CAD (no stenosis)	16.5
1: minimal CAD (1–24% maximal stenosis)	18.4
2: mild CAD (25–49% maximal stenosis)	40.8
3: moderate CAD (50–69% maximal stenosis)	16.5
4: severe CAD (70–99% maximal stenosis)	6.8
5: coronary artery occlusion (100% stenosis)	0.9
Left ventricular global systolic function^b^	
LVEDV (ml)	149.18 ± 23.94
LVESV (ml)	54.64 ± 13.73
LVSV (ml)	94.53 ± 17.16
LVEF (%)	63.46 ± 6.02
Peak of left ventricular myocardial segmental longitudinal strain (%)^b^	
1: basal anterior	16.98 ± 4.02
2: basal antero-septal	17.26 ± 3.87
3: basal infero-septal	17.06 ± 4.26
4: basal inferior	17.95 ± 3.85
5: basal infero-lateral	18.27 ± 3.95
6: basal antero-lateral	17.57 ± 4.32
7: mid-chamber anterior	17.41 ± 4.36
8: mid-chamber antero-septal	17.86 ± 3.58
9: mid-chamber infero-septal	17.57 ± 4.75
10: mid-chamber inferior	16.97 ± 2.14
11: mid-chamber infero-lateral	17.54 ± 4.41
12: mid-chamber antero-lateral	16.77 ± 3.09
13: apical anterior	13.87 ± 2.89
14: apical septal	16.25 ± 3.57
15: apical inferior	14.85 ± 1.98
16: apical lateral	15.69 ± 2.97
Peak of left ventricular myocardial layer longitudinal strain (%)^b^	
Basal	17.38 ± 4.15
Mid-chamber	17.42 ± 3.97
Apical	14.98 ± 2.78
Peak of left ventricular myocardial global longitudinal strain (%)^b^	15.84 ± 3.85

*CAD* coronary artery disease, *CAD-RADS* Coronary Artery Disease-Reporting and Data System, *CCTA* coronary computed tomography angiography, *LVEDV* left ventricular end-diastolic volume, *LVESV* left ventricular end-systolic volume, *LVEF* left ventricular ejection fraction, *LVSV* left ventricular stroke volume

^a^Percentages

^b^Arithmetic mean ± standard deviation

In the study group the mean peak of LV GLS was 15.84 ± 3.85%, including 17.38 ± 4.15% for the basal layers, 17.42 ± 3.97% for the mid-chamber layers and 14.98 ± 2.78% for the apical layers. In the segmental analysis similar strain values were observed for different segments of the myocardium. The lowest value of the peak of LS was found for the anterior apical segment (13.87 ± 2.89%), while the highest value of the peak of LS was found for the infero-lateral basal segment (18.27 ± 3.95%), Table 4.

In a comparative analysis of subgroups differing in the degree of exposure to ETS heart rate during CT acquisition was statistically significantly lower in subgroup A than in subgroup D, Table 5.

**Table 5:** CCTA parameters in the study subgroups

	Subgroups differing in the degree of ETS exposure			
	A: no exposure (*n* = 48)	B: low exposure (*n* = 16)	C: medium exposure (*n* = 21)	D: high exposure (*n* = 18)
Heart rate during CCTA acquisition (bpm)	72.17 ± 9.22^&^	72.63 ± 10.06	75.66 ± 10.54	79.11 ± 12.95
Left ventricular global systolic function				
LVEF (%)	64.38 ± 5.79	64.59 ± 6.01	62.11 ± 5.86	61.55 ± 6.86
Peak of left ventricular myocardial				
Layer longitudinal strain (%)				
Basal	18.95 ± 3.57	19.02 ± 4.54	17.11 ± 4.34	12.05 ± 3.64*
Mid-chamber	19.04 ± 3.38	18.87 ± 3.24	16.89 ± 2.15	12.41 ± 3.89*
Apical	17.12 ± 2.87^&^	13.71 ± 4.09	14.12 ± 5.01	11.29 ± 2.29
Peak of left ventricular myocardial				
Global longitudinal strain (%)	17.35 ± 3.31^&^	15.39 ± 4.58	15.23 ± 3.27	14.21 ± 3.01

	Subgroups differing in the presence of ETS exposures	
	A: no ETS exposure (*n* = 48)	B-D: ETS exposure (*n* = 55)
Heart rate during CCTA acquisition (bpm)	72.17 ± 9.22	75.91 ± 11.34
Left ventricular global systolic function		
LVEF (%)	64.38 ± 5.79	62.65 ± 6.11
Peak of left ventricular myocardial		
Layer longitudinal strain (%)		
Basal	18.95 ± 3.57^#^	16.01 ± 3.18
Mid-chamber	19.04 ± 3.38^#^	16.00 ± 4.03
Apical	17.12 ± 2.87^#^	13.11 ± 4.15
Peak of left ventricular myocardial		
Global longitudinal strain (%)	17.35 ± 3.31^#^	14.94 ± 4.02

Arithmetic mean ± standard deviation

*CCTA* coronary computed tomography angiography, *ETS* environmental tobacco smoke; *LVEDV* left ventricular end-diastolic volume, *LVESV* left ventricular end-systolic volume, *LVEF* left ventricular ejection fraction, *LVSV* left ventricular stroke volume

*A, B, C vs. D: *p* < 0.05

^&^A vs. D: *p* < 0.05

^#^A vs. B–D: *p* < 0.05

In a comparative analysis of subgroups differing in the degree of exposure to ETS peak of LV GLS was statistically significantly lower in subgroup D than in subgroup A. Moreover, a peak of LV basal layer LS and peak of LV mid-chamber layer LS were statistically significantly lower in subgroup D than in subgroups A, B, and C, while peak of LV apical layer LS was statistically significantly lower in subgroup D than in subgroup A.

By comparing the subgroups of patients differing in the fact of exposure to ETS (i.e., subgroup A with subgroup B–D resulting from the combination of subgroups B, C, and D), it was shown that both peak of LV GLS and peak of LV basal layer LS, peak of LV mid-chamber layer LS, and peak of LV apical layer LS, were statistically significantly lower in the subgroup B–D than in the subgroup A. The result of the comparative analysis is presented in Table 5.

**Fig. 1:**
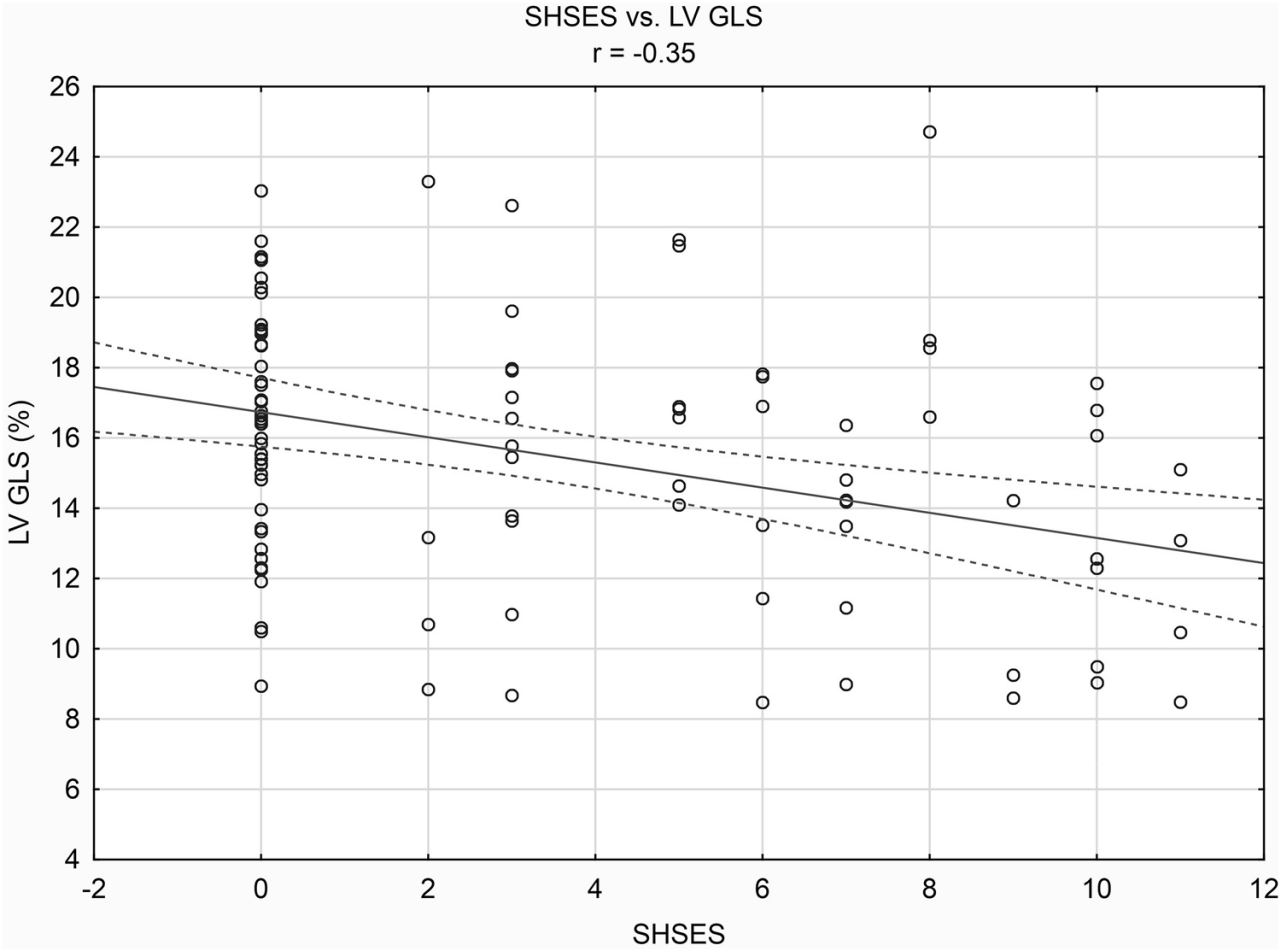
The correlation between SHSES score and LV GLS

The correlation analysis showed the existence of a negative linear relationship between the exposure to ETS expressed by the SHSES scale and peak of LV GLS (*r* = − 0.35, *p* < 0.05). The correlation is presented in Fig. 1. Moreover, negative linear relationships were found between age and LV GLS (*r* = − 0.29, *p* < 0.05), between diastolic blood pressure and LV GLS (*r* = − 0.42, *p* < 0.05), between LVMI in echocardiography and LV GLS (*r* = − 0.47, *p* < 0.05), and between heart rate during acquisition CT and LV GLS (*r* = − 0.37, *p* < 0.05).

In the backward multivariate regression analysis the relationship between potential independent variables, i.e., anthropometric parameters, blood pressure values, efficiency of blood pressure control, parameters of the lipid profile and fasting glucose concentration, used antihypertensive treatment, left ventricular hypertrophy and left ventricular diastolic dysfunction in echocardiography, heart rate during CCTA acquisition, severity of CAD in the CAD-RADS classification and exposure to ETS, and the dependent variable, i.e., LV GLS, was determined. The following statistically significant estimation model was obtained:

peak of LV GLS = 23.635–0.075 age + 1.342 effective blood pressure control + 2.147 β-blockers − 2.852 LVH − 1.773 LVDD − 0.536 CAD-RADS − 0.137 SHSES.

Regression analysis showed that higher SHSES score, higher age, left ventricular hypertrophy, left ventricular diastolic dysfunction, and higher CAD-RADS are independent risk factors for lower peak of LV GLS values. On the contrary, the effective blood pressure control appeared to be independent protecting factor against lower peak of LV GLS values. The results of the regression analysis are listed in Table 6.

**Table 6:** Multivariable stepwise backward regression analysis results

Model for: peak of left ventricular myocardial global longitudinal strain (%)			
	Regression coefficient	SEM of regression coefficient	*p*
Intercept	23.635	1.909	< 0.001
Age (years)^a^	− 0.075	0.028	< 0.01
Effective blood pressure control^b^	1.342	0.534	< 0.05
LVH^b^	− 2.852	0.556	< 0.001
LVDD^b^	− 1.773	0.481	< 0.001
CAD-RADS^a^	− 0.536	0.210	< 0.05
SHSES score^a^	− 0.137	0.054	< 0.05

*CAD-RADS* Coronary Artery Disease-Reporting and Data System, *LVDD* left ventricular diastolic dysfunction, *LVH* left ventricular hypertrophy, *SEM* standard error of mean, *SHSES* Second-Hand Smoke Exposure Scale

^a^Quantitative variables

^b^Dichotomic variables, where 1: yes, 0: no

## Discussion

The conducted study showed that patients with higher ETS exposure had statistically significantly lower LV GLS peak than patients with lower ETS exposure. ETS exposure, expressed quantitatively by SHSES score, was weakly negatively correlated with peak LV GLS. From regression analysis it was shown that a higher SHSES score is an independent risk factor for a lower LV GLS peak. These results complement the current knowledge regarding the relationship between exposure to tobacco smoke and left ventricular function. Study conducted by Yaman et al. indicate that chronic smokers have significantly lower LV GLS assessed by ECHO than non-smokers [14]. This suggest that chronic smokers have LV systolic dysfunction. STE can detect in smokers’ early changes within their heart. The changes are probably consequence of pro-inflammation in cardiovascular system and resulted in different systolic mechanics before its clinical manifestations [15]. Can Bostan et al. assessed heart chamber functions using STE in 70 non-smokers and 80 smokers. Smokers had lower LV GLS than non-smoker control group and it suggests, as authors wrote, impaired myocardial function in the first group. Number of cigarettes smoked each day did not significantly impact on LV GLS. This suggests that even light healthy smokers could have reduced cardiac function [16]. Effects on cardiac function and structure were also studied among people exposed to tobacco smoke in their households and who did not have CAD or severe valvular disease. Past exposure to tobacco smoke and at the time of the study were assessed. ECHO findings were compared with tobacco smoke exposure considering variables such as age, sex, alcohol consumption, study site, and physical activity. Results showed that exposure to tobacco smoke during childhood was associated with lower mitral inflow early-to-late diastolic flow (*E*/*A*) velocity ratio. Exposure during adolescence or adulthood was associated with larger right atrial size, higher LVEF, and lower isovolumetric index. Worse LV GLS occurred in those exposed to tobacco smoke in both childhood and adulthood. The study suggests that exposure to tobacco smoke is associated with deterioration of systolic and diastolic cardiac function. Chronic exposure to tobacco smoke causes oxidative stress, endothelial damage, and increased blood pressure. The pattern of myocardial deformation in chronic tobacco smoke exposure is like early heart failure with preserved ejection fraction (HFpEF), abnormal diastolic function, and myocardial deformation mechanics but normal systolic function [17]. Our study is likely to be the first to find a reduction in left ventricular strain values in people exposed to environmental tobacco smoke using the CT method.

Apart from the main aim of the study, the obtained results indicate the existence of independent relationships between older age and lower LV strain values, between left ventricular hypertrophy and lower LV strain values, between left ventricular diastolic dysfunction and lower LV strain values, between higher severity of coronary artery disease and lower values of the LV strain, and between effective blood pressure control and higher LV strain values. Other researchers have obtained similar relationships so far.

Healthy subjects without cardiovascular disease were shown to have decreasing GLS values with age, e.g., in a study by Stylidis et al. [18]. An analysis of the effect of age on left ventricular longitudinal strain was also made by Alcidi et al. [19]. He evaluated GLS by 2D Doppler echocardiography with quantitative assessment of GLS. GLS decreased with age, although a significant decrease was noted in the age groups 50–60 and above 60 years. The effect of age on GLS remained significant in separate multiple linear regression analyses also after adjustment for confounding factors.

Extensive research on the relationship between left ventricular hypertrophy, left ventricular diastolic dysfunction, and LV GLS in patients with arterial hypertension allowed to establish that GLS is particularly useful in revealing early subclinical LV systolic dysfunction in this group of patients [20]. Galderisi et al. indicated that the basal parts of the interventricular septum are the first segments of the LV myocardium to change under pressure overload [21]. Bendiab et al. documented that in nearly half of patients with arterial hypertension, LV GLS may be decreased [22]. At the same time, they indicated that in patients with preserved LVEF, decreased GLS more often affects patients with long-term and uncontrolled hypertension. Another study by Galdersini et al. indicated a strong association of left ventricular diastolic dysfunction with low LV GLS values, which was independent of changes in afterload and the degree of left ventricular hypertrophy [23].

Reduced GLS in patients with coronary artery disease was demonstrated, among others, by Biering-Sørensen et al. [24], Choi et al. [25], Liou et al. [26], Dhalslett et al. [27] and Antoni et al. [28]. In these studies, various CAD-related endpoints were associated with a statistically significant reduction in GLS: starting with the presence of stenosis ≥ 50% [26, 27], through the presence of stenosis ≥ 70% [24], stenosis of the left main coronary artery, 3-vessels disease [25], and ending with mortality due to acute coronary syndrome [28].

The usefulness of echocardiographic parameters of myocardial strain as good indicators for monitoring the effectiveness of treatment in patients with arterial hypertension was demonstrated by Imbalzano et al. [29]. Similarly, Niu et al. showed that myocardial strain parameters assessed by magnetic resonance can be a good marker for long-term monitoring of the effectiveness of treatment in patients with hypertensive heart disease [30]. At the same time, they indicated that GRS and GCS are more sensitive to treatment; it is difficult to improve GLS impairment in patients with hypertensive heart disease.

Yingchoncharoen et al. conducted meta-analysis to estimate normal values of GLS, GRS, and GCS. The normative GLS, GRS, and GCS values in echocardiography were as follows: − 19.7% (95% CI − 20.4% to − 18.9%), 47.3% (95% CI 43.6% to 51.0%), and − 23.3% (95% CI − 24.6% to − 22.1%) [31]. In the studied group of patients, relatively low values of peak of left ventricular longitudinal strain are noticeable, especially low values of peak of left ventricular myocardial apical layer longitudinal strain in group D, amounting to 11.29 ± 2.29%. Potential causes of this surprising result in the group of patients with hypertension exposed to ETS include advanced age, poor blood pressure control, coexistence of relatively high heart rate values, left ventricular hypertrophy, left ventricular diastolic dysfunction and possibly significant coronary artery disease in the subjects. Moreover, it should be remembered that the peak strain values are underestimated in the computed tomography assessment in relation to the echocardiographic assessment. In the recently published literature standardizing the CT-derived myocardial strain measurement, it has been shown that computed tomography significantly underestimates the strain values relative to echocardiographic assessment [11]. Ammon et al. showed that the highest predictive accuracy for the echocardiographic GLS < 18% criterion is characterized by the computed tomography-derived GLS < 12% criterion [32].

Our study has some limitations. There is small non-randomized study group. However, the applied statistical tests considered such a group size. The coexistence of many recognized factors lowering the left ventricular strain in the study group is another significant limitation. Non-effective blood pressure control, high heart rate, left ventricular hypertrophy, left ventricular diastolic dysfunction, and significant coronary artery disease have been associated with a reduction in left ventricular strain in many studies. The influence of potentially modifying factors on the studied relationship “exposure to ETS vs. LV GLS” was verified by regression analyses. The studied relationship is independent of other potentially influencing factors. Yet, another limitation in the study group is the absence of a non-hypertensive group. The control group (with normal blood pressure values, not exposed to ETS) among patients recruited to the project to some extent may be patients with a score of 0 on the SHSES scale, with effective blood pressure control assessed based on ABPM. Efficiency blood pressure control as well as systolic blood pressure and diastolic blood pressure were considered in the regression analysis as potential variables influencing the assessed relationship. An important methodological limitation of the study is the use of the CT-derived strain as a method of myocardial contractility assessment instead of the echocardiographic method. The use of echocardiographic strain evaluation is obviously better documented in the literature than the use of CT-derived strain. Strain evaluation by echocardiography is also used in practice more often than by CT. However, the popularity of the CT-derived strain method is growing, and the methodology itself is standardized, as shown by recently published studies [33]. Studies from recent years indicate high repeatability of strain assessment in computed tomography, also high compliance with echocardiographic assessment or magnetic resonance imaging assessment [12, 34]. In our study the reliability of the CT-derived strain evaluation was achieved by performing all tests on the same CT machine according to exactly the same image acquisition protocol, using a high-quality dedicated application for strain evaluation of a recognized company, evaluating the strain strictly according to the application instructions by an experienced researcher with practical skills certified by internationally recognized scientific societies, the use of manual correction of the initial automatic contour of endocardium and epicardium as well as an internal assessment of the repeatability of the assessment by determining the intra-observer and inter-observer variability. In terms of methodology, the lack of determination of blood cotinine concentration in the blood may also be considered a disadvantage of the study. Therefore, the reliability of our respondents who completed the SHSES questionnaire had to be assumed.

## Conclusion

There is an unfavorable weak relationship between environmental tobacco smoke exposure estimated using the Second-Hand Smoke Exposure Scale and computed tomography-derived left ventricular global longitudinal strain in hypertensive patients.

## Data Availability

The data presented in this study are available upon request from the corresponding author. The data are not publicly available.
